# Supplementary material for: TP53-dependent toxicity of CRISPR/Cas9 cuts is differential across genomic loci and can confound genetic screening
Source: Nat Commun. 2022 Aug 4;13:4520. doi: 10.1038/s41467-022-32285-1 (PMC9352712; doi:10.1038/s41467-022-32285-1)
Supplement: Supplementary file 1 — Supplementary Information [file 41467_2022_32285_MOESM1_ESM.pdf]

SUPPLEMENTARY MATERIAL for Álvarez, Biayna and Supek (2022) Nature Communications.

Supplementary Tables.

Supplementary table 1. AUROC measuring how accurate is the discrimination between two sets of essential and non-essential genes. It includes the bootstrap p-value of comparing AUROC between TP53wt and TP53-/- (1-tailed test TP53wt < TP53-/-; no adjustments were made for multiple comparisons). Source data are provided as a Source Data file

treatment	time	TP53	AUROC	p-value
woDOX	*9*	*wt*	0.8453	0.3431
		KO	0.8508	
	12	*wt*	0.8638	0.4574
		KO	0.8651	
	15	*wt*	0.8713	0.3041
		KO	0.8775	
DOX	*9*	*wt*	0.8344	0.7879
		KO	0.8229	
	12	*wt*	0.8480	0.4913
		KO	0.8483	
	15	*wt*	0.8568	0.7319
		KO	0.8486	
DOX+ATRi	*9*	*wt*	0.8221	0.2520
		KO	0.8320	
	12	*wt*	0.8412	0.3526
		KO	0.8466	
	15	*wt*	0.8605	0.6904
		KO	0.8540	

Supplementary table 2. AUROC table using public dataset from Brown et al 2019[1], in the same manner as Supplementary table 1. These analyses included 615 out of 684 essential genes[2], and 726 out of 927 non-essential genes[3] (1-tailed test TP53wt < TP53-/-; no adjustments were made for multiple comparisons). Source data are provided as a Source Data file.

time	replicate	TP53	AUROC	p-value
**9**	A	wt	0.8887	
	B	wt	0.8913	
**12**	A	wt	0.9082	
	B	wt	0.9113	
**15**	A	wt	0.9093	
	B	wt	0.9212	
**18**	A	wt	0.9189	0.9951
		KO	0.8877	
	B	wt	0.9212	0.9991
		KO	0.883	

Supplementary table 3. Table of genes considered to be selected in all or none of the studied samples,

depending on the TP53 status. Although the absence of gene TP53 in the third column is unexpected, the reason for this is that in one sample the beta score was not considered significantly positive due to the FDR = 0.285, slightly above the threshold.

Negatively selected systematically		Positively selected systematically	
**Exclusively in TP53wt**	**Exclusively in TP53-/-**	**Exclusively in TP53wt**	**Exclusively in TP53-/-**
ABHD17A	*CCND3*	*ARRDC3*	*C8orf37*
ASH2L	*DMAP1*	*BCL6*	*DEFB128*
AURKA	*PLRG1*	*C16orf58*	*DPF2*
CBWD3	*POU2F1*	*CDKN2C*	*GUCY2F*
CDCA8	*SARS*	*CDSN*	*PLA2G5*
CGB5	*SNAPC4*	*CYLC2*	*RGP1*
CLTC		*DDIT4*	*SAV1*
COG4		*FAM135A*	*SLC35F6*
CSNK1A1		*HSDL2*	*SMIM9*
DHDDS		*KCTD5*	*TAF1L*
DHX9		*KLF5*	*TBX6*
EIF3I		*LATS2*	*UTP14C*
FAM25G		*MAPK1*	*VNN3*
GFER		*MBNL1*	*ZNF260*
GINS3		*NACC1*	
GOLGA6A		*OR6C70*	
GOLGA6C		*PCDHA7*	
GOLGA6L4		*PGRMC2*	
GOLGA8H		*QKI*	
GTF2A2		*RIC8B*	
H3F3A		*SOX4*	
HCFC1		*SPTBN1*	
HIRA		*SRGAP1*	
HUWE1		*STK24*	
KIAA1429		*TAOK1*	
KRT17		*TCF4*	
MAD2L2		*UBE2A*	
MDM2		*UBE2K*	
MDM4		*VCAM1*	
METAP1			
NCL			
NOB1			
NPIPB6			
NUP214			
PCYT1A			
PES1			
PRPF4B			
PSMA3			
PSMD3			
PWP2			
RAD51			
RCL1			
RHBDF1			
RNF113A			
RPF1			
RPP30			
RPS29			
SENP6			
SKA2			
SRSF3			
SUPT4H1			
SYS1			
TBC1D3B			
TICRR			
TUBA1B			
USP17L19			
USP17L25			
USP17L29			
USP39			
USP7			
ZNF626			

Supplementary table 4. GO terms enriched (p <1e-3) with 57 out of the 61 genes that are systematically negatively selected exclusively in TP53wt samples. In bold, the GO terms related to cell cycle; in bold and italics, the GO terms related to DNA damage response. FDR adjustment accounts for multiple comparisons. n=18,157 total genes, of which 57 are set genes, included in the analysis. Source data are provided as a Source Data file.

GO Term	Description	p-value	FDR	Enrichment	Pathway genes	Set genes in pathway
GO:0090304	nucleic acid metabolic process	5.70E-08	8.90E-04	3.35	2188	23
GO:0043170	macromolecule metabolic process	1.17E-07	9.14E-04	2.06	5884	38
GO:0070646	protein modification by small protein removal	2.59E-07	1.35E-03	9.95	288	9
GO:0006396	RNA processing	1.10E-06	4.29E-03	5.07	817	13
GO:0006807	nitrogen compound metabolic process	1.34E-06	4.20E-03	1.85	6705	39
GO:0016579	protein deubiquitination	1.95E-06	5.07E-03	9.4	271	8
GO:0044238	primary metabolic process	2.57E-06	5.72E-03	1.78	7168	40
GO:0007030	Golgi organization	2.69E-06	5.25E-03	15.17	126	6
GO:0042176	regulation of protein catabolic process	2.95E-06	5.12E-03	7.43	386	9
GO:0006139	nucleobase-containing compound metabolic process	3.20E-06	4.99E-03	2.68	2733	23
GO:0016070	RNA metabolic process	3.97E-06	5.63E-03	3.44	1575	17
GO:1901991	**negative regulation of mitotic cell cycle phase transition**	4.32E-06	5.62E-03	10.52	212	7
GO:0044237	cellular metabolic process	4.68E-06	5.62E-03	1.74	7316	40
GO:0010948	**negative regulation of cell cycle process**	5.93E-06	6.61E-03	8.09	315	8
GO:0008152	metabolic process	6.41E-06	6.68E-03	1.66	8046	42
GO:1901988	**negative regulation of cell cycle phase transition**	6.96E-06	6.79E-03	9.78	228	7
GO:0007049	**cell cycle**	7.49E-06	6.88E-03	6.62	433	9
GO:0046483	heterocycle metabolic process	9.15E-06	7.94E-03	2.52	2905	23
GO:0044260	cellular macromolecule metabolic process	9.94E-06	8.17E-03	2.07	4616	30
GO:0071704	organic substance metabolic process	1.12E-05	8.71E-03	1.69	7539	40
GO:0006725	cellular aromatic compound metabolic process	1.20E-05	8.93E-03	2.48	2952	23
GO:0051301	**cell division**	2.78E-05	1.97E-02	6.53	390	8
GO:0065003	protein-containing complex assembly	3.45E-05	2.34E-02	3.44	1295	14
GO:0045930	**negative regulation of mitotic cell cycle**	4.08E-05	2.66E-02	7.43	300	7
GO:1901360	organic cyclic compound metabolic process	4.09E-05	2.56E-02	2.31	3178	23
GO:0051726	**regulation of cell cycle**	4.62E-05	2.77E-02	3.59	1155	13
GO:0006364	rRNA processing	4.82E-05	2.79E-02	9.14	209	6
GO:0070647	protein modification by small protein conjugation or removal	4.87E-05	2.72E-02	3.85	994	12
GO:0034641	cellular nitrogen compound metabolic process	5.23E-05	2.81E-02	2.27	3226	23
GO:0009894	regulation of catabolic process	5.31E-05	2.77E-02	3.81	1003	12
GO:0000278	**mitotic cell cycle**	6.95E-05	3.50E-02	11.63	137	5
GO:0006508	proteolysis	7.51E-05	3.66E-02	3.42	1211	13
GO:1903311	regulation of mRNA metabolic process	8.66E-05	4.10E-02	6.6	338	7
GO:0010564	**regulation of cell cycle process**	8.75E-05	4.02E-02	4.32	737	10
GO:0006974	***cellular response to DNA damage stimulus***	1.00E-04	4.46E-02	4.25	749	10
GO:0016072	rRNA metabolic process	1.01E-04	4.39E-02	8	239	6
GO:0090230	regulation of centromere complex assembly	1.44E-04	6.08E-02	106.18	6	2
GO:0034470	ncRNA processing	1.59E-04	6.55E-02	5.98	373	7
GO:0010972	**negative regulation of G2/M transition of mitotic cell cycle**	1.90E-04	7.62E-02	14	91	4
GO:0034660	ncRNA metabolic process	2.38E-04	9.28E-02	4.8	531	8
GO:0034622	cellular protein-containing complex assembly	2.43E-04	9.24E-02	3.81	835	10
GO:1901990	**regulation of mitotic cell cycle phase transition**	2.72E-04	1.01E-01	5.48	407	7
GO:0042770	***signal transduction in response to DNA damage***	2.95E-04	1.07E-01	12.49	102	4
GO:0043933	protein-containing complex subunit organization	2.98E-04	1.06E-01	2.82	1584	14
GO:0045786	**negative regulation of cell cycle**	3.01E-04	1.05E-01	4.63	550	8
GO:0018205	peptidyl-lysine modification	3.05E-04	1.04E-01	6.52	293	6
GO:0061136	regulation of proteasomal protein catabolic process	3.05E-04	1.01E-01	8.47	188	5
GO:1902750	**negative regulation of cell cycle G2/M phase transition**	3.06E-04	9.96E-02	12.37	103	4
GO:0001649	osteoblast differentiation	3.42E-04	1.09E-01	12.02	106	4
GO:0003283	atrial septum development	3.44E-04	1.07E-01	70.79	9	2
GO:0006397	mRNA processing	3.63E-04	1.11E-01	5.22	427	7
GO:0030163	protein catabolic process	3.63E-04	1.09E-01	5.22	427	7
GO:0031329	regulation of cellular catabolic process	4.14E-04	1.22E-01	3.57	893	10
GO:1901987	**regulation of cell cycle phase transition**	4.41E-04	1.27E-01	5.06	441	7
GO:1903047	**mitotic cell cycle process**	6.19E-04	1.76E-01	4.16	613	8
GO:1903050	regulation of proteolysis involved in cellular protein catabolic process	6.26E-04	1.75E-01	7.24	220	5
GO:0044267	cellular protein metabolic process	6.42E-04	1.76E-01	2.03	3293	21
GO:0032434	regulation of proteasomal ubiquitin-dependent protein catabolic process	6.77E-04	1.82E-01	10.03	127	4
GO:0007346	**regulation of mitotic cell cycle**	6.81E-04	1.80E-01	4.1	622	8
GO:0006977	***DNA damage response; signal transduction by p53 class mediator resulting in cell cycle arrest***	6.85E-04	1.78E-01	17.38	55	3
GO:1902400	***intracellular signal transduction involved in G1 DNA damage checkpoint***	7.23E-04	1.85E-01	17.06	56	3
GO:1902403	***signal transduction involved in mitotic DNA integrity checkpoint***	7.23E-04	1.82E-01	17.06	56	3
GO:1902402	***signal transduction involved in mitotic DNA damage checkpoint***	7.23E-04	1.79E-01	17.06	56	3
GO:0072431	***signal transduction involved in mitotic G1 DNA damage checkpoint***	7.23E-04	1.76E-01	17.06	56	3
GO:0072413	**signal transduction involved in mitotic cell cycle checkpoint**	7.23E-04	1.74E-01	17.06	56	3
GO:0016071	mRNA metabolic process	7.72E-04	1.83E-01	4.02	634	8
GO:0009057	macromolecule catabolic process	8.41E-04	1.96E-01	3.26	978	10
GO:0016925	protein sumoylation	9.28E-04	2.13E-01	15.67	61	3
GO:0008380	RNA splicing	9.55E-04	2.16E-01	5.25	364	6
GO:0006511	ubiquitin-dependent protein catabolic process	9.69E-04	2.16E-01	4.42	504	7
GO:0044265	cellular macromolecule catabolic process	9.92E-04	2.18E-01	3.48	824	9

Supplementary table 5. List of candidate ATRi-sensitizing genes identified in TP53wt samples.

**genes**	**related function**
CKS2, CKS1B	cyclin-dependent protein kinase activity
ETAA1, PIF1, DCLRE1C	DNA repair
CAPRIN1, ATP5C1, OR1E1	Other

Supplementary Figures.

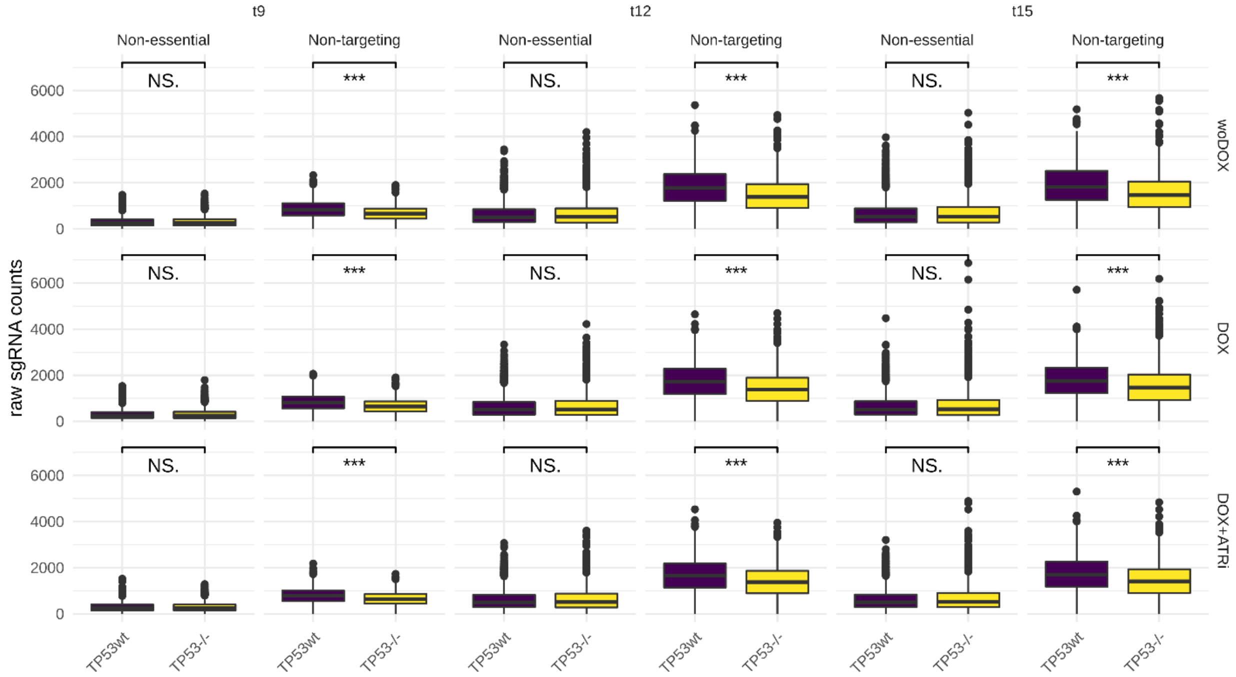

Supplementary figure 1. Pooled counts of two sgRNA sets (non-essential genes and non-targeting sgRNAs). sgRNA counts are median-normalized to allow sample comparison. Test: two-tailed Mann-Whitney. *** denotes a *p*-value <9e-11. No adjustments were made for multiple comparisons. n=4,580 independent sgRNAs examined over six independent experiments at three time points. Source data are provided as a Source Data file.

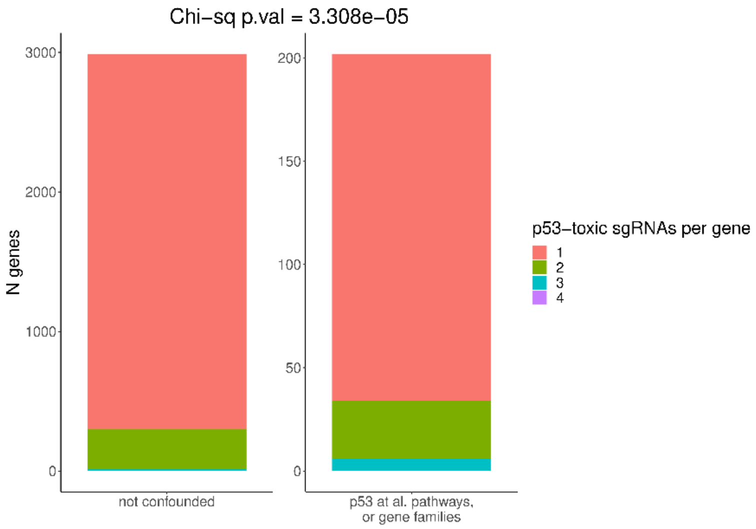

Supplementary figure 2. Among the genes containing at least one sgRNA target whose DSB is especially toxic in TP53wt, the confounded genes (right; i.e., those whose effect is probably due to gene function loss – top-50 p53 interactors, and genes in other enriched pathways – or to sequence similarities within the gene family) contain significantly more putatively p53-toxic sgRNAs than the not confounded genes (left). Test: Pearson's Chi-square, p=3.3e-5. n=3,192 genes examined. Source data are provided as a Source Data file.

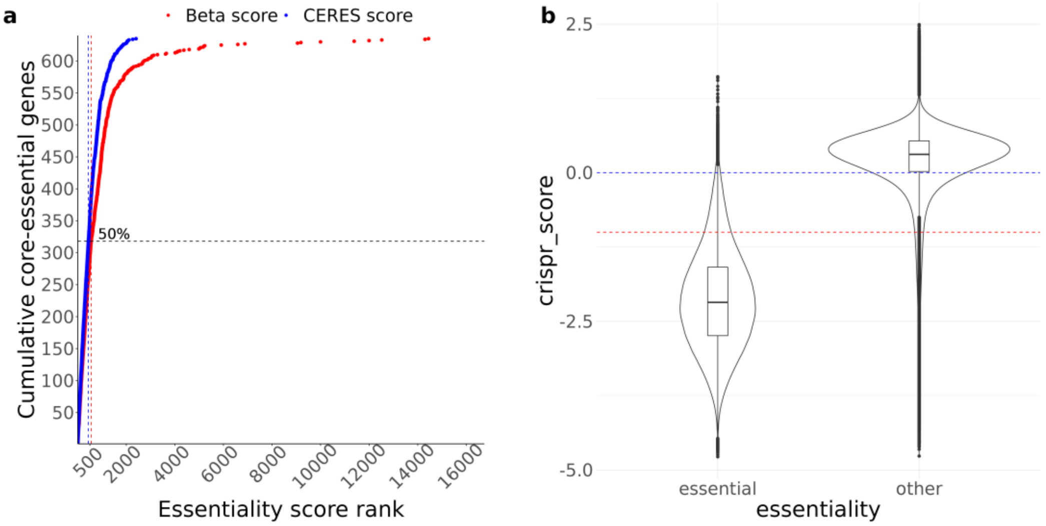

Supplementary figure 3. a) Genome-wide rank of core-essential genes according to two essentiality scores: mean MAGeCK-MLE beta score (red) or mean CERES score (blue). Vertical dashed lines indicate the rank of the 50th percentile core-essential gene (i.e. the 318th, horizontal dashed line) in each case. The Essentiality score rank (x axis) consists of the overlapping genes between Brunello and Avana libraries ranked from more to less essential according to each score. b) Comparison of CRISPR scores for all pooled cell lines and genes, obtained from the combined Achilles and PScore studies[5]. A score < -1 (below red dashed line) indicates gene essentiality. The left boxplot includes only the set of core-essential genes considered in our analyses[2], while the boxplot on the right are all the remaining genes. n=17,486 independent sgRNAs examined over 906 cell lines. Source data are provided as a Source Data file.

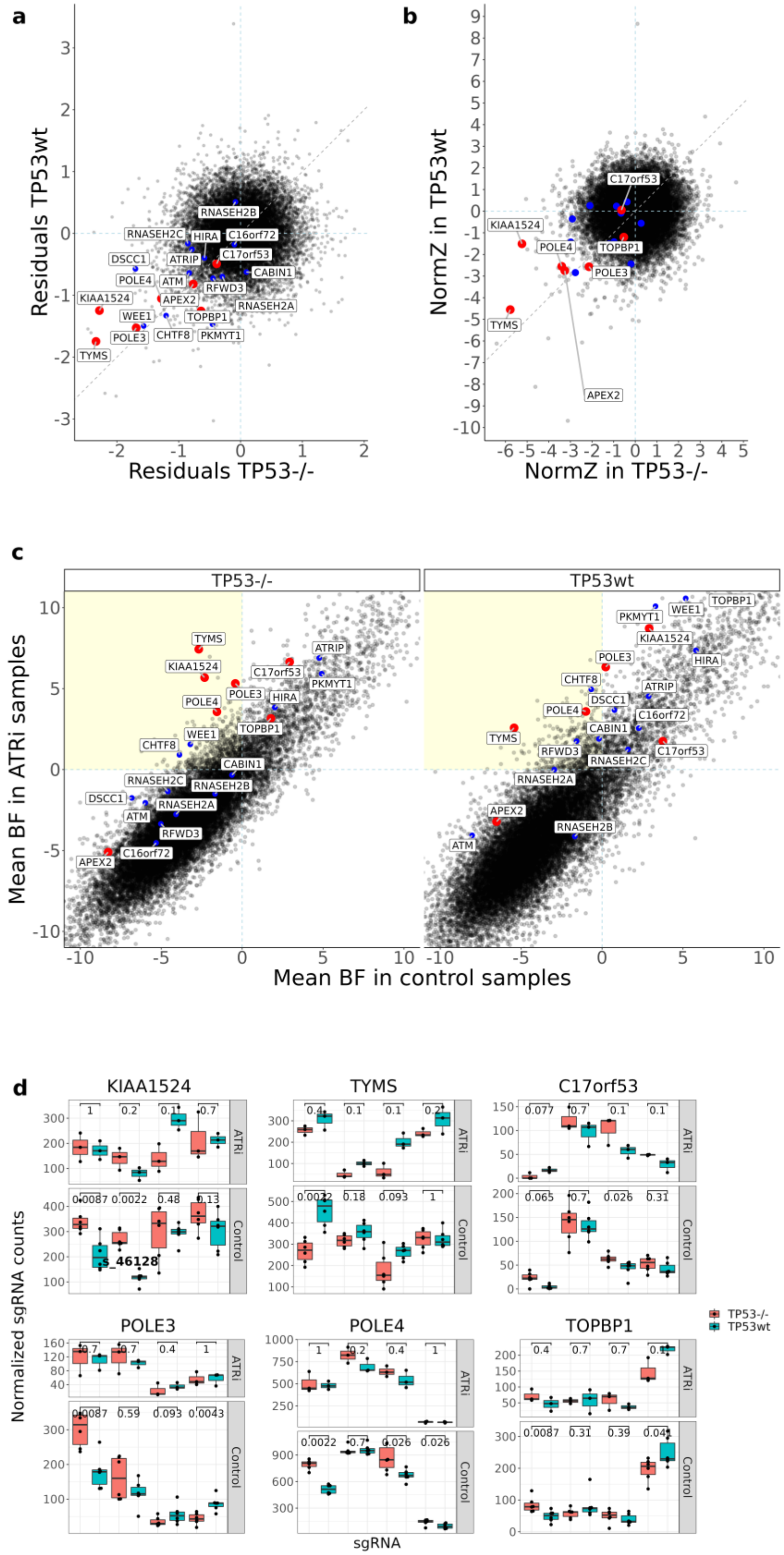

Supplementary figure 4. a) Comparison of the regression residuals from the two plots in Figure 1E, thus genes above the x = y line (gray) have a stronger signal in TP53-/-. b) Analysis of conditional selection

upon ATRi using the drugZ statistical methodology. Negative *normZ* values suggest conditional essentiality. Genes above the x = y line (gray) have a stronger signal in TP53-/-. Five of the top-7 known ATR inhibitors genes have more negative normZ scores in TP53-/- than in TP53wt. c) Analysis usign BAGEL v2. Upper-left quadrant (light yellow) contains genes with potential ATRi-dependent essentiality (mean Bayesian Factor >0 in ATRi-treated samples and =<0 in control samples). In TP53-/- samples, four of the top-7 genes are found essential in ATRi-treated samples but not in the controls, while only two fulfil this condition in TP53wt samples. d) Normalized sgRNA counts (y-axis) for the six top-7 genes that are replicated in A549. The x-axis represents the four sgRNAs that target each gene. Each dot is a sample at a time point 9, 12, or 15, and Control includes untreated and DOX-treated: therefore, n=6 samples at Control and n=3 at ATRi boxplots. Tests: 2-tailed Mann-Whitney. Source data are provided as a Source Data file.

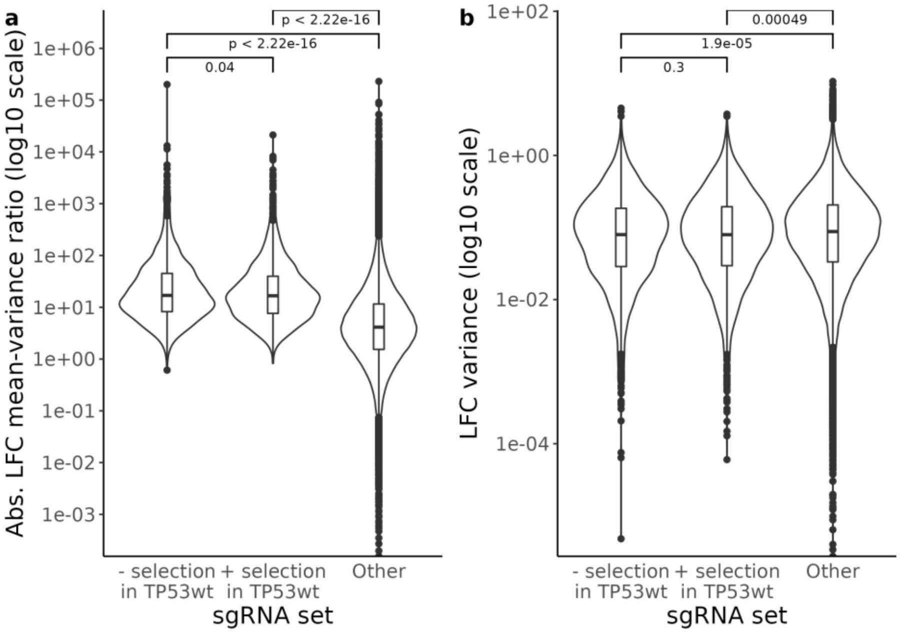

Supplementary figure 5. LFC mean:variance (left) and variance (right) distributions for the different sgRNA sets. a) Absolute log_2 fold-change (LFC) mean-variance ratio of each sgRNA between the three pseudo-replicate screens on the A549 cell line (y-axis). sgRNAs that are truly negatively selected in TP53wt compared to TP53-/- will show higher absolute ratios, as opposed to false positives. Indeed, the negatively selected sgRNA set (first element of x-axis; *target loci* in the main text) has a significantly higher absolute LFC mean-to-variance ratio than the corresponding positively selected set (second element of x-axis; Tests: 1-tailed Mann-Whitney, p = 0.04). b) Same dataset as in panel 5A, but showing instead the distribution of sgRNA LFC variance instead of the LFC mean-variance ratio. n=77,441 independent sgRNAs examined (2,879 in - selection in TP53wt, 2,786 in + selection in TP53wt, 72,031 in Other). Source data are provided as a Source Data file.

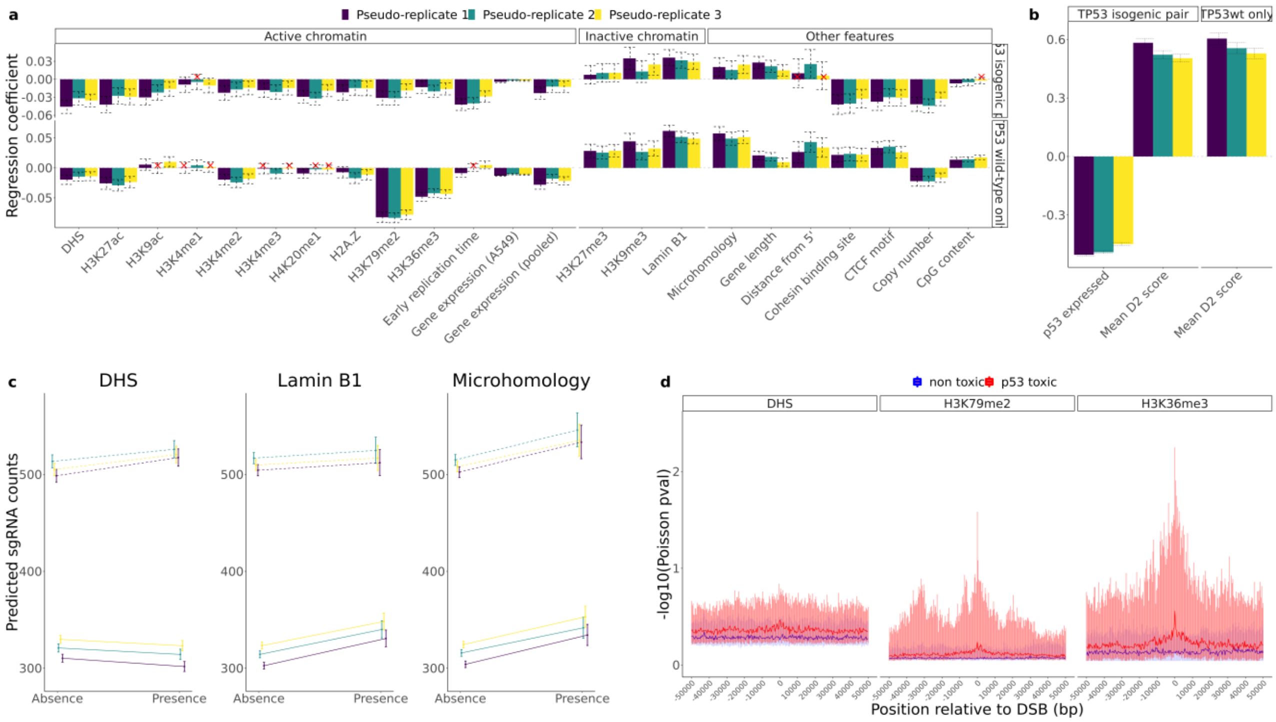

Supplementary figure 6. Enrichment of various chromatin features next to toxic break loci, as estimated by the NB regression coefficients. a) NB regression coefficients, expanding the results shown in Figure 2A (namely, the bottom panel including only the TP53wt samples, and additional features). Each colour represents one of the three pseudo-replicates. TP53 isogenic pair indicates interaction of TP53 with a given variable, while TP53 wild-type only indicates the effect of each variable per se regressed against the same sgRNA set, including only the TP53wt samples (see Materials and Methods). Negative regression coefficients indicate a decrease of sgRNA counts. Each chromatin feature regressed independently. Red crosses indicate FDR>0.25. n=10,050 independent sgRNAs and 23 chromatin features examined over six (top panel) or three (bottom panel) samples, at three time points. Dashed error bars represent the SE of the mean. b) Effect of p53 and gene essentiality (D2 score) per se on cut toxicity of target and background loci. Error bars represent the SE of the mean. c) Regressions also represented in Figure 2B, here showing absolute values. Dashed lines indicate TP53-/- samples, while full lines represent TP53wt samples. n=10,050 independent sgRNAs and three chromatin features examined over six independent experiments. Error bars represent the 95% CI of the mean. No adjustments were made for multiple comparisons. d) Results for the analysis detailed in Figure 2C but using the averaged -log10(Poisson p-value) ChipSeq score. Source data are provided as a Source Data file.

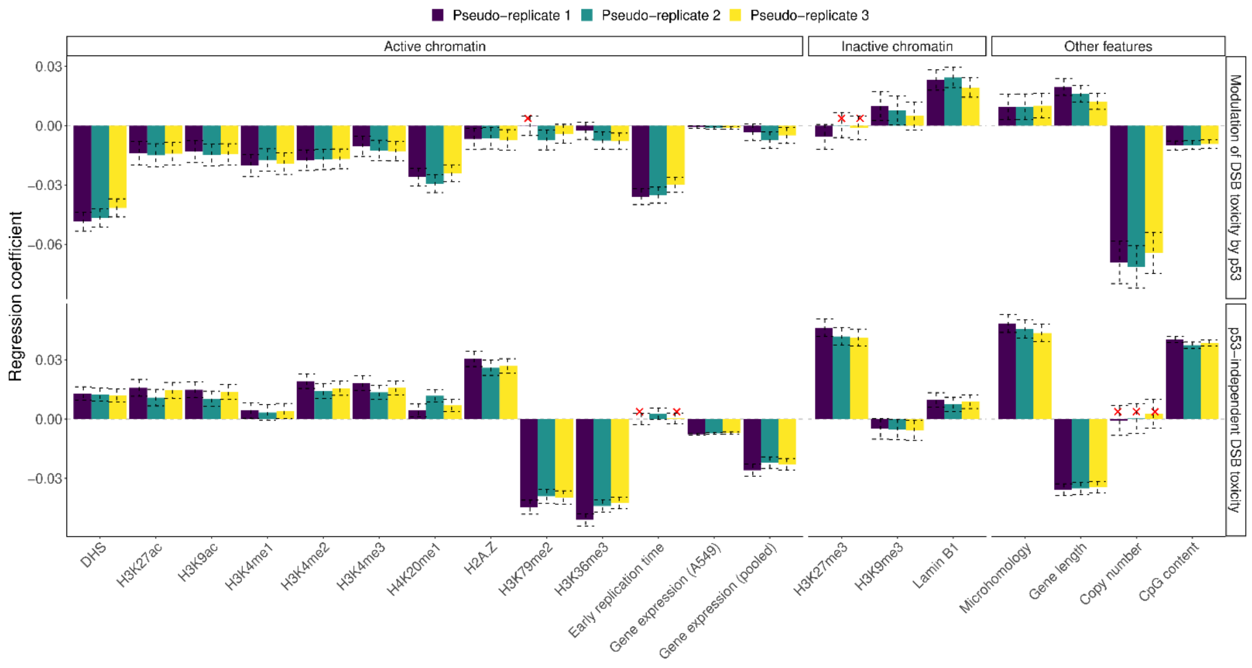

Supplementary figure 7. A comparison contribution of chromatin features to general (bottom) and p53-related (top) DSB toxicity, using all sgRNAs from Brunello library. To estimate the (relative) magnitude of the p53-dependent versus p53-independent toxicity of DSB, we ran an analogous analysis in which all sgRNAs from the Brunello library were included, instead of just the TP53-dependent subset (target loci). This allows one to capture other potential sources of DSB toxicity. The bottom barplot is an estimate of the DSB toxicity at a given chromatin feature without the participation of p53. Meanwhile, the top barplot represents the contribution of p53 activity to the DSB toxicity. Negative values denote a toxic effect, and vice versa. The p53-independent toxicity applies to gene body-resolution features: active transcription mark H3K79me2 and H3K36me3 (toxic) and high expression levels (toxic), and the Polycomb silencing mark H3K27me3 (protective). The p53-dependent toxicity applies more to chromosomal domain-resolution features of active chromatin such as early replication time (p53-toxic), the lack of heterochromatin mark H3K9me3 (protective against p53-toxicity), and a higher copy number (p53-toxic, as above). Lamin B1 and DNA sequence microhomology are protective both generally and with regards to p53-specific toxicity. By virtue of our experimental design that involves a TP53 isogenic cell line pair, we are more confident about the effect size estimates for the p53-dependent toxicity features (where the experiment is stringently controlled) than for the p53-independent toxicity component (which may be confounded by multiple factors). Error bars represent the SE. n=55,662 independent sgRNAs and 20 chromatin features examined over six independent samples at three time points. Source data are provided as a Source Data file.

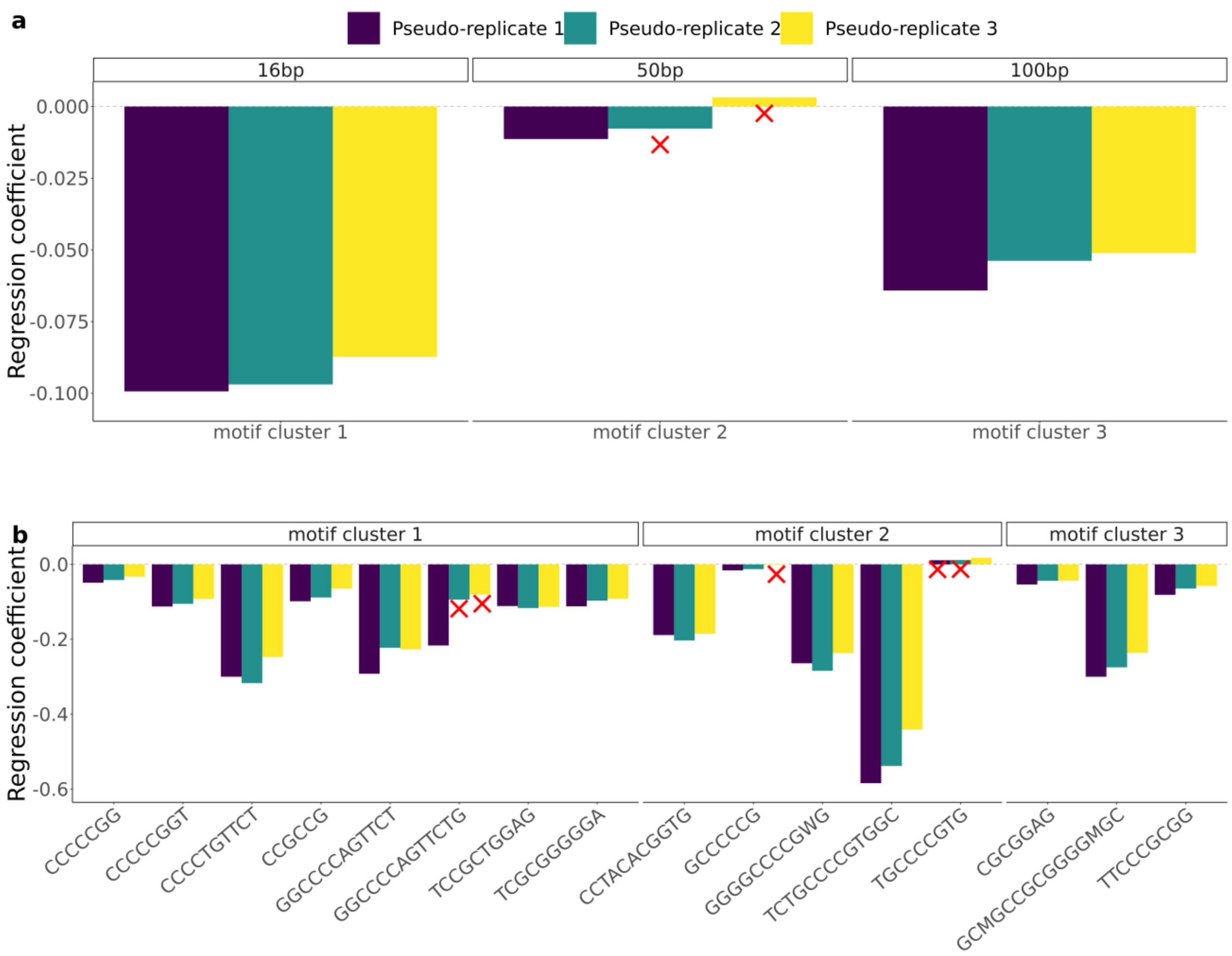

Supplementary figure 8. DNA sequence motifs that are enriched at high-p53-toxicity sgRNA target sites. Each colour represents one of the three pseudo-replicates. Regression coefficients indicate the effect of each motif regressed against the same sgRNA set as in Figure 1D-E, but including only the TP53wt samples. Negative estimates indicate a decrease of sgRNA counts. a) The three motif clusters combine similar motifs identified by HOMER as enriched in target loci (FDR<1e-5) – at different genomic ranges surrounding the sgRNA cut position – that also show a significant (FDR<0.25; red crosses indicate FDR>0.25) and consistent association with higher p53 toxicity in the NB regressions. b) Separate regression coefficients for all the motifs that are combined by similarity into either of the three motif clusters. Source data are provided as a Source Data file.

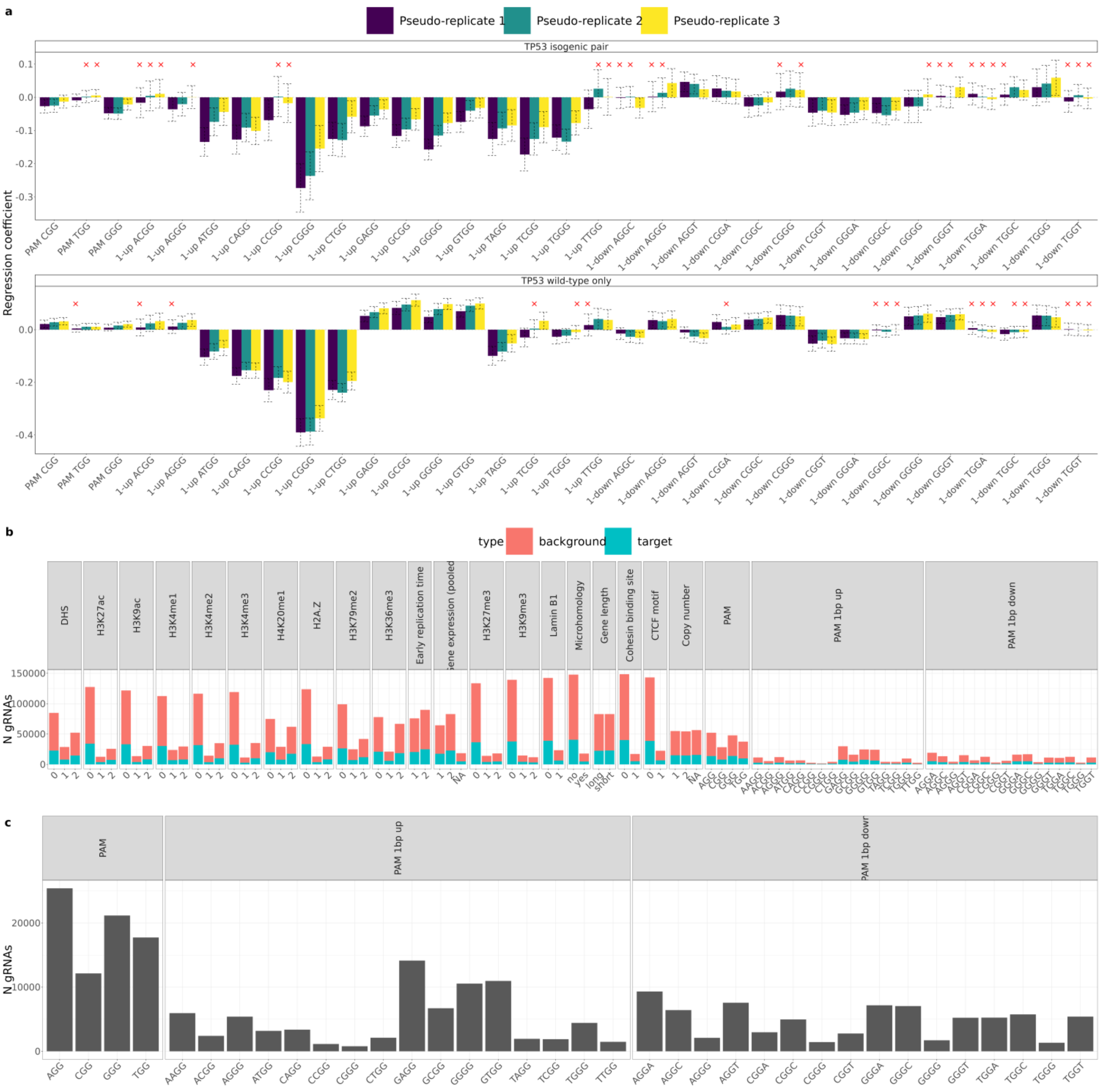

Supplementary figure 9. DNA sequence motif analyses. a) Estimates of all PAM sequence context regressions. Each colour represents one pseudo-replicate. TP53 isogenic pair indicates interaction of TP53 with a given variable, while TP53 wild-type only indicates non-interacting variables regressed against the same sgRNA set, but including only the TP53wt version of each pseudo-replicate. Negative estimates indicate a decrease of sgRNA counts. Red crosses indicate FDR>0.25. Error bars represent the SE. n=10,050 independent sgRNAs, four PAM motifs, and 32 PAM motif contexts, examined over six independent samples at three time points. b) Total PAM sequence frequencies in Brunello library. c) Frequencies of chromatin feature bins and PAM sequences in the target and background sgRNAs. 5'-

CNGG-3’ is one of the least frequent patterns in the 5’-NNGG-3’ PAM tetranucleotide context, both when only the target and background sequences are considered, as well as for the full sgRNA library: also, the target-to-background sequence ratio is larger for 5’-CNGG-3’ than for the other 5’-NNGG-3’ patterns, indicating an enrichment of target sequences that contain this pattern. The NA category includes those sgRNAs that were not included in the considered bins, or that there was no available data for them. Source data are provided as a Source Data file.

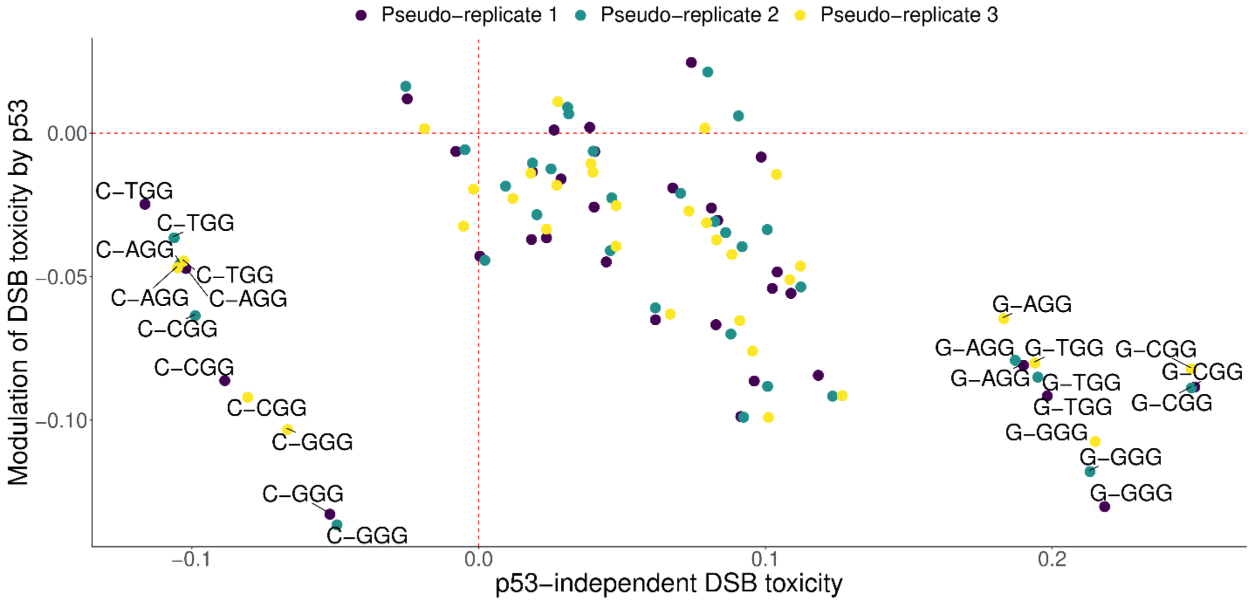

Supplementary Figure 10. Contribution of PAM sequence to general (x-axis) and p53-related (y-axis) DSB toxicity. x-axis: fitness effect (negative values denote a toxic effect, and vice versa) of a Cas9 double strand break (DSB) in TP53-/- cells. y-axis: PAM:*TP53* interaction term coefficient, which estimates how the DSB toxicity is altered in TP53 wild-type compared to TP53-/- cells. Source data are provided as a Source Data file.

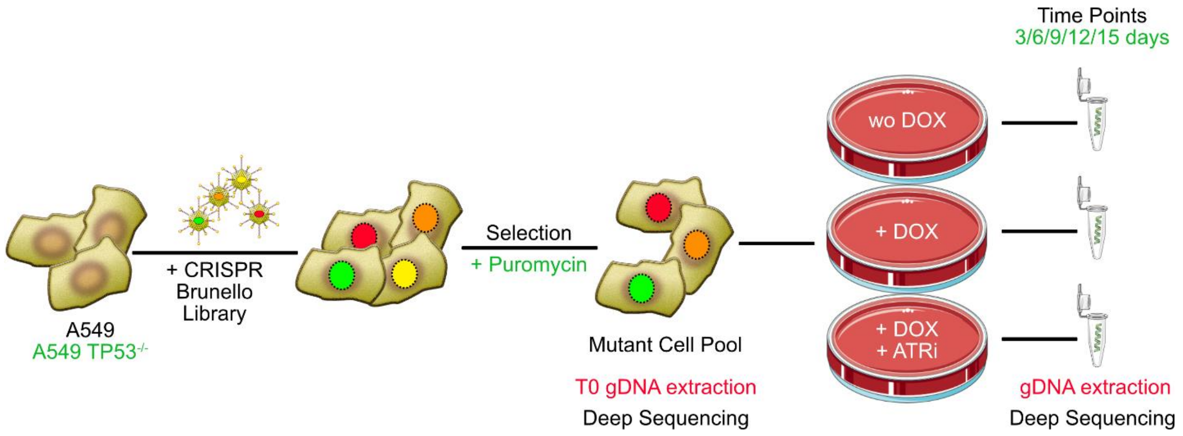

Supplementary figure 11. Schematic representation of the experimental workflow for CRISPR-Cas9 screens. Screens used Brunello library and were performed in lung adenocarcinoma cell line A549. The

experimental data for the “w/o DOX” and “+ DOX” conditions (which we here treated as pseudoreplicates) is from our previous publication Biayna *et al*.[6]; the data on the ATRi treated condition is particular to the current study. This figure was partially made using Servier Medical Art (smart.servier.com, accessed on 11 July 2022, CC-BY 3.0).

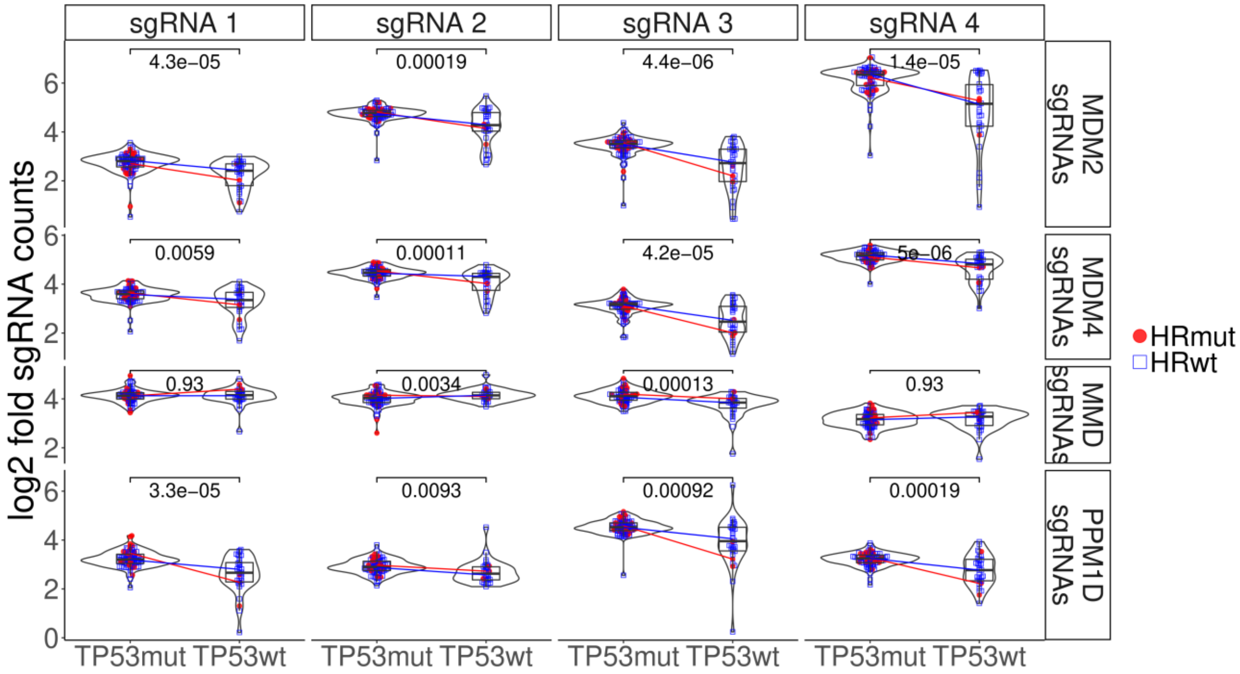

Supplementary figure 12. Association study across cell line panels suggest roles of DNA repair pathways in Cas9 DSB toxicity. Example of how the target loci from the Avana data set were ascertained, showing *MDM2* and *MDM4* genes as reference – since they have an expected functional interaction with *TP53* –, and *MMD* and *PPM1D* as the genes containing the three top target loci. To be considered a target locus, a given sgRNA is firstly required to have a significantly lower read count in TP53wt cell lines (see Materials and methods). As expected, almost all *MDM2* and *MDM4* sgRNAs pass this criterion, while this is only fulfilled by sgRNA 3 from *MMD*, and sgRNA 1 and 4 from *PPM1D*. Lines connect the median read counts of HRmut (red) or HRwt (blue) cell lines. 2-tailed T-test p-values are shown. n=124 independent cell lines (19 TP53mut-HRmut, 71 TP53mut-HRwt, 3 TP53wt-HRmut, and 31 TP53wt-HRwt). Source data are provided as a Source Data file.

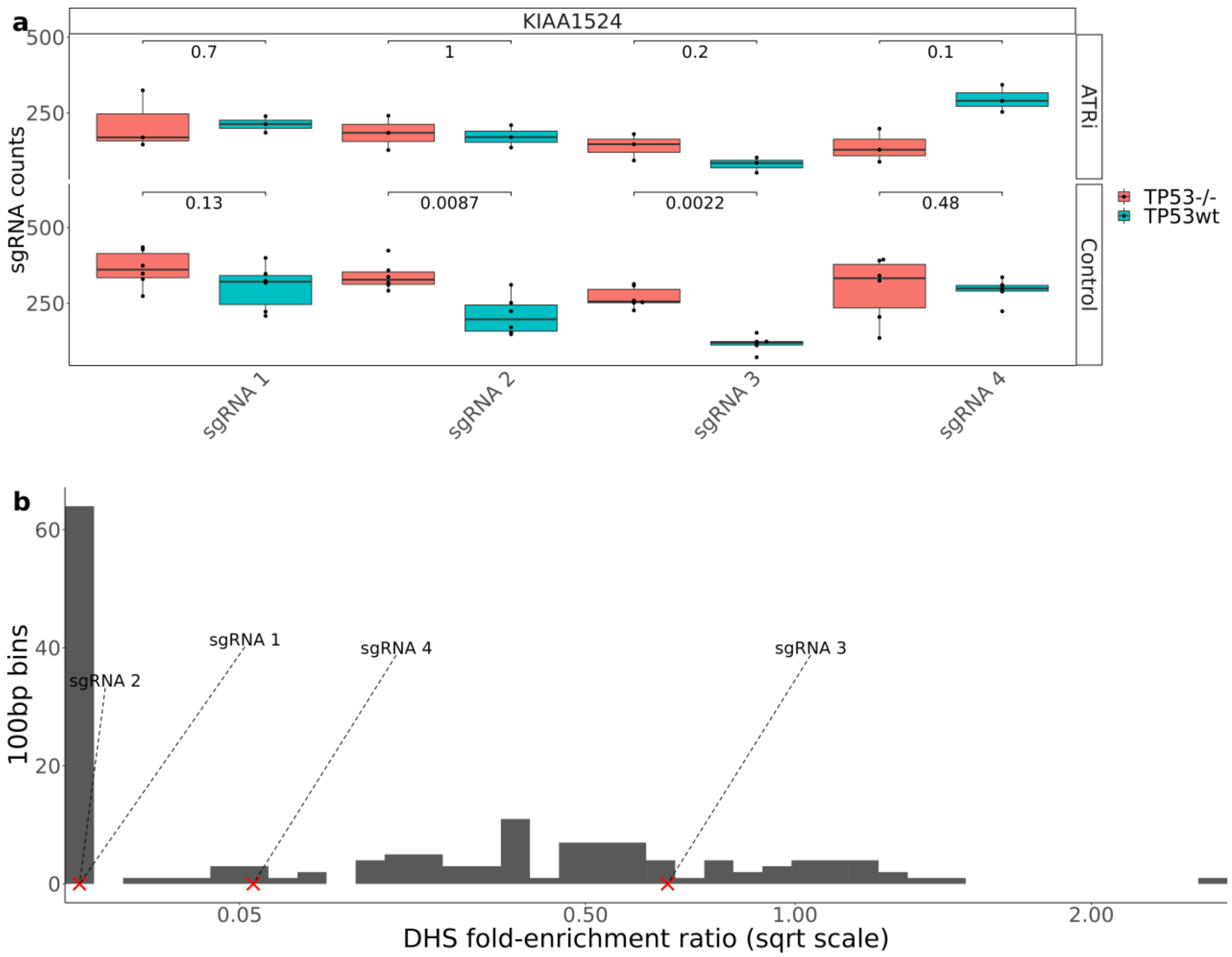

Supplementary figure 13. a) Normalized sgRNA counts for *KIAA1524*/*CIP2A*'s four sgRNAs. The sgRNA number 3 is different from the rest in that it exhibits a significant (p=0.0022) count drop in TP53wt compared to TP53-/-. Each dot is a sample at a time point 9, 12, or 15; Tests: 2-tailed Mann-Whitney. n=2 independent ATRi-treated samples at three time points, and n=4 independent control samples at three time points. b) Abundance of DHS represented as the ChipSeq fold-enrichment ratio (x-axis, square root scale), averaged at each 100bp bin genomic position within *KIAA1524*/*CIP2A*. y-axis represents the abundance of 100bp bins for each fold-enrichment ratio interval. Red crosses mark the fold-enrichment ratio for the 100 bp bin where each sgRNA (labelled) is located. Source data are provided as a Source Data file.

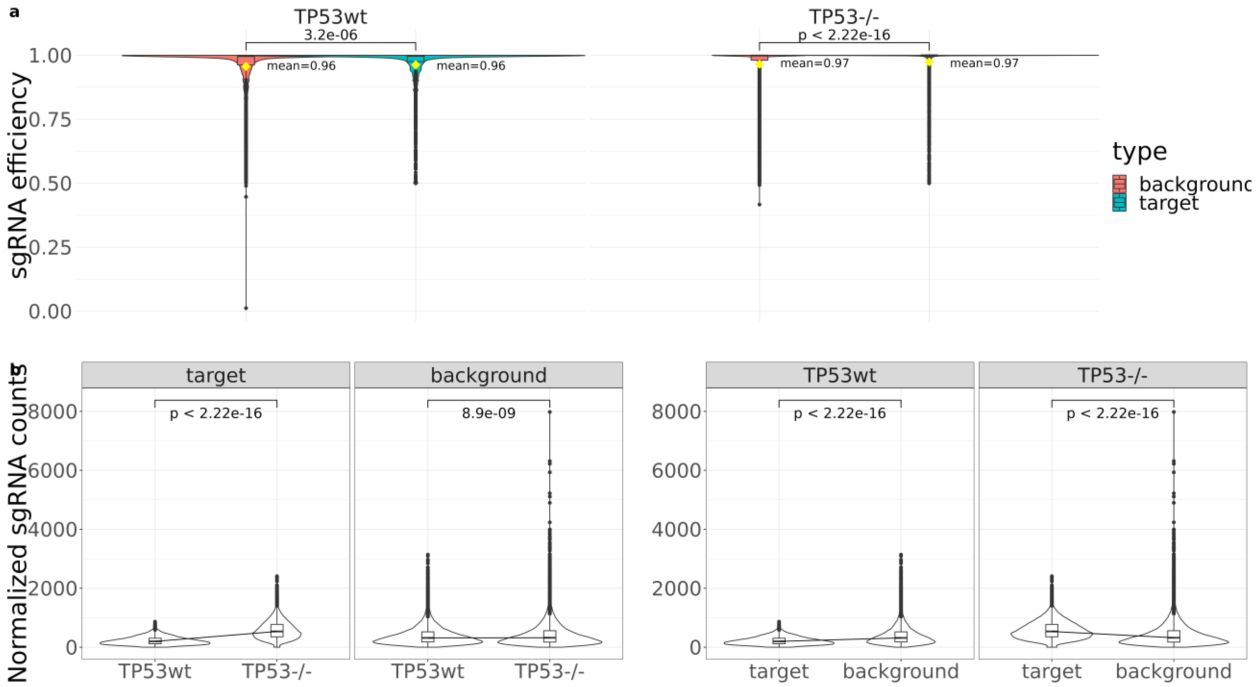

Supplementary figure 14. Analyzing whether lower Cas9 efficiency in heterochromatin could be causing the apparent increment of p53-mediated toxicity in euchromatin. a) median MAGeCK-MLE estimated sgRNA efficiency in background and target sequences (y-axis) for each *TP53* genotype (x-axis); yellow rhombus=mean, 1-tailed Mann-whitney test p-values shown above the violin plots; p=3.2e-6 and p<2.22e-16 for TP53wt and TP53-/-, respectively). b) Normalized sgRNA counts (y-axis) for background and target sequences in each *TP53* genotype (x-axis and facets). 2-tailed Mann-Whitney test p-values shown above the violin plots. n=10,554 independent sgRNAs examined over six independent samples (three TP53wt and three TP53-/-) at three time points.

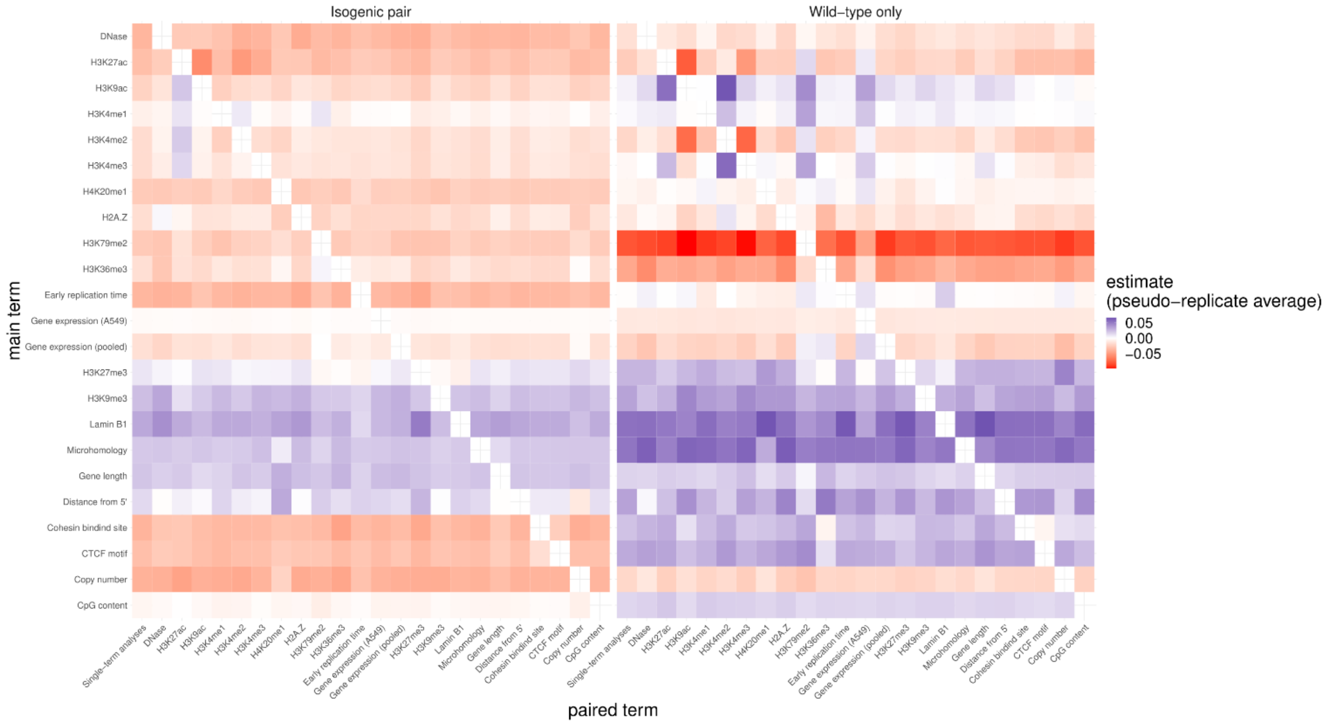

Supplementary figure 15. Paired term regression analyses. Regression coefficient estimates for each feature (main term) when another feature (paired term) is included in the regression formula. Estimates are averaged across the four sample replicates. Single-term analyses refers to the original estimate without a paired term, as in Figure 2A and Supplementary figure 6A.

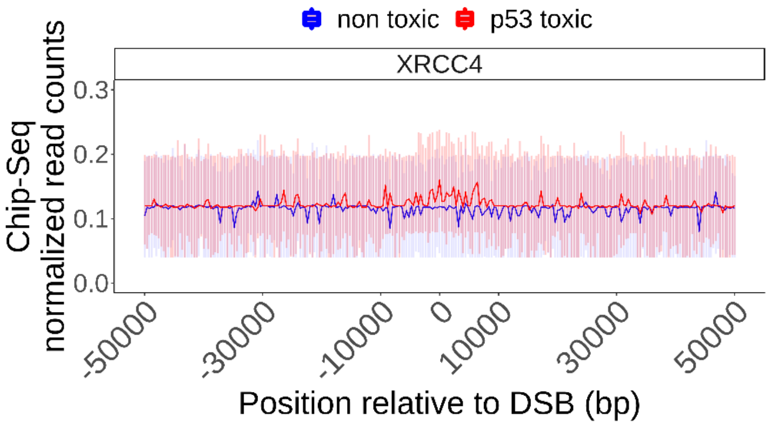

Supplementary figure 16. **Chip-Seq normalized read count data for the key NHEJ protein XRCC4**. The plot shows that XRCC4 peaks around the cut position of the p53-toxic compared to the non-p53-toxic sgRNAs. This plot is analogous to Figure 2C (in brief, normalized read counts were averaged at each 400bp-bin position relative to the sgRNA cut position (0), including the top 200 target loci showing more p53-related DSB toxicity – larger negative LFC, red – and top 200 non-selected loci – LFC closer to 0, blue. Vertical lines represent the 25-75% interquantile range at each bin, and left-to-right lines connect the medians). Source data are provided as a Source Data file.

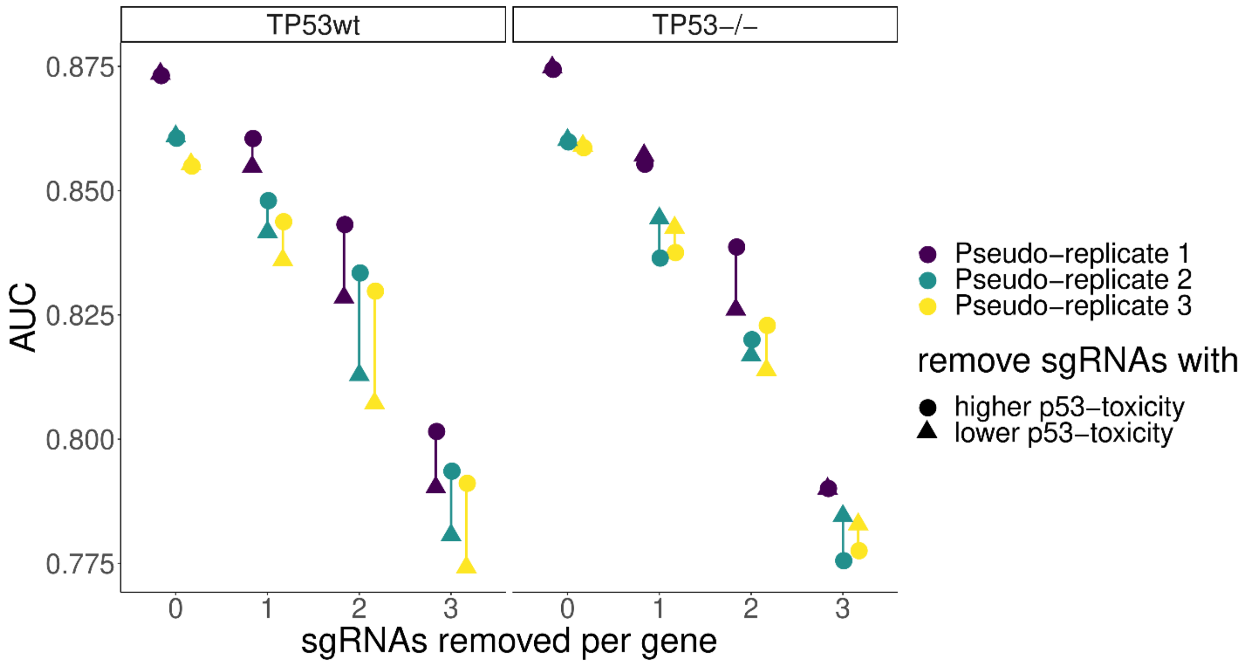

Supplementary figure 17. **Differential decrease of AUC (y-axis) upon removal of count data from one or more sgRNAs per gene (x-axis)**. Circles indicate that the removed sgRNAs were the ones with higher p53-toxicity, and triangles indicate the opposite. Source data are provided as a Source Data file.

Supplementary Text.

Supplementary Text 1 – A *TP53* wild-type background can confound estimates of gene selection in genetic screens

a) Overlap of confounded negative selection with public datasets. We ran an additional analysis to explore the overlap of genes negatively selected exclusively in TP53wt samples between various genome-wide CRISPR screening libraries. For each gene, the exact sites targeted by the sgRNAs in different libraries commonly differ, allowing to uncouple the gene-level fitness effects from sgRNA locus-level effects. Screening data for the RPE1 human normal cell line (retinal pigment epithelium) *TP53*-isogenic pairs was obtained from two studies that used the sgRNA libraries Brunello[7,8] and Gecko v2[9,10]. For each study, we ran a MAGECK-MLE analysis as for our A549 lung cancer isogenic pair (see Materials and methods), identifying genes that are negatively selected in the TP53wt but not in the TP53-/- cells. As expected, the pairwise gene overlap is highest between replicates from the same study (Figure 1d). Notably, the overlap between our A549 data and the RPE1 study that employed the same Brunello sgRNA library ($\log_2$OR = 0.19 and 0.2 for the two A549 pseudo-replicates; see Methods for definition of overlap) is comparable to (and even slightly larger than) the overlap between the two RPE1 studies, which use different libraries ($\log_2$OR = 0.19 and 0.14 for Gecko Replicates 1 and 2, with the Brunello library); Figure 1D. In other words, the concordance between experiments using the same library in different cell lines is similar or larger than the concordance between different libraries on the same cell line. This is compatible with a mechanism where the differential p53-mediated DSB toxicity underlies our observations. A plausible explanation is that because the sgRNAs of different libraries target different loci within the same gene, this could elicit different DSB toxicity and thus fitness effects, even though their effects on the function of the targeted gene would presumably be similar.

b) Top-7 ATRi-sensitizer gene *KIAA1524/CIP2A* is not recovered in TP53wt, and it contains one p53-toxic sgRNA. *KIAA1524/CIP2A* is the only top-7 gene that is recovered in TP53-/- and missed in TP53wt when using drugZ and BAGEL algorithms, and also when using a slightly more stringent beta score threshold in MAGeCK-MLE. Interestingly, *KIAA1524/CIP2A*'s 3rd sgRNA is the only within the top-7 genes that is among the target loci (see previous sections), and it indeed shows a significant (p=0.0022) count drop in TP53wt compared to TP53-/- (see panel A of the Supplementary figure 13, and Supplementary figure 4D for other top-7 genes): this could potentially bias the *KIAA1524/CIP2A* sgRNA counts of TP53wt samples towards negative values in the control samples, thus removing the difference between control and ATRi-treated samples. Importantly, and in line with the results above, this sgRNA differs from the other sgRNAs of *KIAA1524/CIP2A* in that it targets a DHS region (see Supplementary figure 13, panel b).

c) Discovery of putative A549-specific ATRi-sensitizing genes is hampered in TP53wt. We asked if we can detect previously unreported genes that are synthetic lethal with *ATR* inhibition in the A549 lung adenocarcinoma cell line. Using MAGeCK-MLE, we identified genes that were conditionally essential in ATRi-treated vs. untreated beta score comparisons at different time points (days 9, 12, and 15) and using two pseudo-replicates (see Materials and methods), yielding a total of six independent comparisons per *TP53* status. Analyses employing TP53-/- samples identified two putative ATRi-sensitizing genes: *FBXW7* (substrate recognition component of a SCF E3 ubiquitin-protein ligase) and *ATP5H* (a mitochondrial ATP-synthase). They were synthetic lethal with *ATR* inhibition in four out of six TP53-/- comparisons, while they

were not detected in any TP53wt comparison. Interestingly, *FBXW7* is a common tumor suppressor gene[11], mutated in ~2.5% non-small cell lung cancers[12] (a cancer type matching the A549 cell line), and additionally in >5% of endometrial, colon, small intestine, bladder, head-and-neck and cervical cancer. Our analyses suggest that loss-of-function *FBXW7* mutations may sensitize to ATRi, however also that this is contingent upon the genetic background and/or cell type (since *FBXW7* was not listed among the common ATRi-sensitizing genes in other cell lines[13]). In addition, eight genes were found to be synthetic lethal with ATRi in at least four out of the six comparisons when using TP53wt samples (see Supplementary table 5), however none were among the top-7 known genes, in contrast to three of the top-7 genes - *POLE3*, *TYMS*, and *KIAA1524/CIP2A* - thusly recovered in TP53-/-; this again supports the notion that the TP53wt background impedes discovery of synthetic lethal genes.

Supplementary Text 2 – Association of active chromatin marks with high-p53-toxicity sgRNA target sites

a) Off-targeting enrichment at high-p53-toxicity sgRNA target sites. We considered the hypothesis that multiplicity of cuts due to off-targeting might drive higher p53 toxicity[14]. We therefore computed the total Cutting frequency determination[8] (CFD) score for each target or non-selected locus with *CRISPRseek*[15]. We identified an enrichment of off-target effects (see Materials and methods) in only 425 (13%) target loci, which consist on the overlap of 364 (11%) target loci that have significantly higher CFD than the observed in the non-selected loci, and 236 (7%) for which there exist one or more exact sequence matches across the genome -- for comparison, there were 92 (2%) such loci among the non-selected. In addition, two Mann-Whitney tests confirm both higher CFD and amount of exact matches per sgRNA in the target loci distribution, compared to the non-selected ($p = 2.44e-12$ and $p < 2.2e-16$, respectively). Since this enrichment of potential multi-targeted sgRNA effect could potentially account for a small part of the library-dependent p53 toxic effect, we removed these 425 sgRNAs from subsequent analyses.

b) Analyzing whether lower Cas9 efficiency in heterochromatin could be causing the apparent increment of p53-mediated toxicity in euchromatin. If the increment of p53-mediated toxicity when DSB are introduced into euchromatin were due to the lower Cas9 efficiency in heterochromatin, we would expect to see lower sgRNA efficiency in the background than in the target sequences (panel A in Supplementary figure 14). However, for each TP53 genotype (x-axis) the median MAGeCK-MLE estimated sgRNA efficiency (y-axis) does not differ substantially between sgRNA sets: mean (yellow rhombus) and median efficiencies are >0.9 in all cases, although there is a slightly lower median efficiency in background sgRNAs. Likewise, target sequences would be more negatively selected in TP53-/- compared to the background, due to higher rates of gene function loss and general DSB-associated toxicity (panel B in Supplementary figure 14). However, this panel shows that target sequences are not negatively selected in TP53-/- compared to the background (2-tailed Mann-Whitney test p-values shown above the violin plots; see especially the rightmost comparison, $p<2.22e-16$).

c) Paired term regression analyses. We considered the regression coefficient estimates for each feature (main term) when another feature (paired term) is included in the regression formula (Supplementary figure 15). Among others, the modest effect of DSB distance from 5' gene end disappears when correcting for gene length; MH loses the association when correcting for H4K20me1; Lamin B1 has a more protective

effect when correcting for H3K27me3; Gene expression loses the association when H3K79me2 or CN are included; H2A.Z loses the association when DHS is included; H3K27ac increases the association when correcting for H3K9ac; H3K27me3 has a less protective effect when H3K9me3 is included; and H3K36me3 loses the association when H3K79me2 or CN are included.

d) Support for NHEJ increasing p53-related toxicity of DSB. To provide more evidence that NHEJ increases the p53-related toxicity of DSB, we initially considered running an analysis analogous to that employed in the case of HR repair (drawing on HR-deficient cancer cell lines). However, this is not feasible, since NHEJ deficiencies are not recognized to occur in cancers nor cancer cell lines. Thus it is not clear whether the occurrence of mutations in NHEJ-related genes (e.g. *XRCC4*) is an effective proxy of NHEJ inactivation. Therefore, we followed a different approach to shed some light on the matter. Firstly, there is recent evidence that H3K4 methylation promotes NHEJ by blocking end resection. Our data (top barplot in Supplementary figure 6a) shows that there is increased p53-related DSB toxicity in H3K4-methylated regions, consistent with a higher p53-related DSB toxicity of NHEJ repair. Furthermore, the same study claims that H3K4me3 as opposed to H3K4me1 is the main promoter of *RIF1* accumulation (which contributes to blocking end resection); our above-mentioned analysis (top barplot in Supplementary figure 6a) shows that p53-related DSB toxicity increases with the methylation levels (H3K4me1 lowest, H3K4me2 and H3K4me3 highest). Overall, this association with histone marks supports that NHEJ repair triggers more p53-related toxicity than mechanisms that rely on DNA resection, such as HR and MMEJ. Secondly, it has been stated that, generally, damage in active chromatin undergoes preferential repair via HR. Taking this into consideration, our result showing that active chromatin is less toxic by itself (Supplementary Figure 7, bottom panel) would be in accordance with a higher toxicity of NHEJ. Finally, Chip-Seq normalized read count data for the key NHEJ protein XRCC4[16] shows a peak of this protein around the cut position of the p53-toxic compared to the non-p53-toxic sgRNAs; see the Supplementary figure 16. The positive association of XRCC4 Chip-Seq signal with p53-toxic sgRNA targets provides direct evidence for NHEJ involvement, while the association with genomic features such as H3K4me3 is further consistent with it (although alone constitutes circumstantial evidence).

e) Quantitative guidelines for the design of sgRNAs that are less affected by the TP53 status of the cells. The criteria we found to influence p53-related toxicity of DSB could be summarized into a convenient quantitative score. To do so, we have assigned a p53-toxicity score to every sgRNA in the widely-used Brunello, Avana, TKO, and Gecko libraries. This score consists of a combination of several of the most relevant variables associated with such toxicity: presence of Lamin B1 and microhomology (MH), binarized (as described in Materials and methods) GC content, cutting frequency determination (CFD), abundance of DHS, H3K9me3, copy number (CN), and replication time (RT).
The contribution of each feature to the p53-toxic phenotype score is the exponentiated regression coefficient of its interaction with TP53 status, averaged across the three A549 pseudo-replicates. This bears some resemblance with the analysis shown in Supplementary figure 7, with some distinctions: i) no sgRNA filtering based on the Cutting Frequency Determination (CFD; measure of the off-targeting effect of a sgRNA target sequence) score was applied, and ii) all the interaction terms were run in the same regression, thus ensuring that each association is conditioned upon the other factors (which may be correlated), i.e.

```
sgRNA counts ~ DHS*TP53status + H3K9me3*TP53status + RT*TP53status +
LaminB1*TP53status + CN*TP53status + MH*TP53status + GCcontent*TP53status +
CFD*TP53status + D2score + offset
```

Please note that we also included in the regression above the features i) presence of a C upstream of the PAM, and ii) H3K79me2 abundance. However, their interaction coefficients were not <0 (i.e. no toxic effect in this particular analysis, possibly due to correlations with other features) and were thus not included in the final score.

feature	contribution (exp(regression coefficient))
CN	0.941
DHS	0.970
GC content	0.979
RT	0.979
CFD	0.998
H3K9me3	1.01
Lamin B1	1.01
MH	1.01

A contribution < 1 implies a toxic effect (lower sgRNA counts) for the presence/abundance of a given feature, and *vice versa*. The p53-toxicity score for each sgRNA was calculated as follows:

$$\text{p53-related DSB-toxicity score} = \Pi_{\text{features}}\ \text{bin} \times (\text{contribution} - 1) + 1$$

where a feature's bin is either 0 or 1. The scores were then rescaled so that the most p53-toxic sgRNA across libraries is assigned a 1, and the least p53-toxic one is assigned a 0.

To gauge the accuracy of this empirical p53 toxicity score, we assigned the corresponding score to each sgRNA in Brunello library, and compared the distributions between the sets of sgRNAs that are strongly negatively (target loci in text) or positively selected in TP53 wild-type compared to TP53-/- cells, or not strongly selected to either side. Mann-Whitney one-tailed tests (p-value = 1.7e-13) show that the known negatively selected set of sgRNA has higher p53 toxicity phenoscores than normal sgRNAs, and the positively selected set has the lowest scores (p<2.22e-16), so this score seems to correctly capture the p53-toxicity triggered by DSB.

However, since the Brunello library is not specifically designed to measure the variation in toxicity of DSB, we think it would be important to have further research involving an custom sgRNA library to interrogate the effects of various chromatin states, so that a more comprehensive classifier can be developed.

f) Quantitative estimation of the decrease in sensitivity when sequentially removing one to three sgRNAs from a CRISPR screening, taking into account the predicted p53-related DSB-toxicity of the targeted genomic locus. We made use of the A549 screening samples from our recent study Biayna et al. PLOS Biology 2021 (three pseudo-replicates, using mean gRNA counts between time points 9, 12, and 15). Namely, we calculated the AUC (for identifying known essential genes; see Materials and methods) using the full library (Brunello), and then also sequentially removing one, two, and up to three sgRNAs per gene, and checked the effect that this had on the AUC (i.e. the ability to differentiate core-essential from non-essential genes). The removed sgRNAs were either (a) the ones with the highest predicted p53 toxicity, or (b) the one with the lowest predicted p53-related DSB toxicity, according to a custom prioritization score that we defined in Supplementary Text 2E. Expectedly, the results show that, regardless of TP53 status, there is better accuracy if there are more sgRNAs targeting a gene. Importantly, the decrease in accuracy tends to be ameliorated if removing sgRNAs with the higher p53-toxicity according to our score (circle-shaped points), and is exacerbated when the removed sgRNAs have the lowest p53-toxicity (triangle-

shaped points). This effect is only found in TP53wt, also as hypothesized: in particular, in the case of TP53wt the mean AUC for the three pseudo-replicates after removing one, two, or three sgRNAs with the highest predicted p53-toxicity is 0.828, but if the removed sgRNAs are the ones with the lowest p53-toxicity the mean AUC is 0.814; in the case of TP53-/- the mean AUC in both scenarios is 0.818. Overall, this supports that the higher-toxicity sgRNAs have higher potential of confounding identification of essential genes in genetic screens, especially in a TP53wt environment. See Supplementary figure 17.

REFERENCES

1. Brown, K. R., Mair, B., Soste, M. & Moffat, J. CRISPR screens are feasible in TP53 wild-type cells. *Molecular Systems Biology* **15**, (2019).
2. Hart, T. *et al.* Evaluation and Design of Genome-Wide CRISPR/SpCas9 Knockout Screens. *G3 Genes|Genomes|Genetics* **7**, 2719–2727 (2017).
3. Hart, T., Brown, K. R., Sircoulomb, F., Rottapel, R. & Moffat, J. Measuring error rates in genomic perturbation screens: gold standards for human functional genomics. *Mol Syst Biol* **10**, 733 (2014).
4. Meyers, R. M. *et al.* Computational correction of copy number effect improves specificity of CRISPR–Cas9 essentiality screens in cancer cells. *Nat Genet* **49**, 1779–1784 (2017).
5. Pacini, C. *et al.* Integrated cross-study datasets of genetic dependencies in cancer. *Nat Commun* **12**, 1661 (2021).
6. Biayna, J. *et al.* Loss of the abasic site sensor HMCES is synthetic lethal with the activity of the APOBEC3A cytosine deaminase in cancer cells. *PLoS Biol* **19**, e3001176 (2021).
7. Haapaniemi, E., Botla, S., Persson, J., Schmierer, B. & Taipale, J. CRISPR–Cas9 genome editing induces a p53-mediated DNA damage response. *Nature Medicine* **24**, 927–930 (2018).
8. Doench, J. G. *et al.* Optimized sgRNA design to maximize activity and minimize off-target effects of CRISPR-Cas9. *Nature Biotechnology* **34**, 184–191 (2016).
9. Sanjana, N. E., Shalem, O. & Zhang, F. Improved vectors and genome-wide libraries for CRISPR screening. *Nat Methods* **11**, 783–784 (2014).
10. Drainas, A. P. *et al.* Genome-wide Screens Implicate Loss of Cullin Ring Ligase 3 in Persistent Proliferation and Genome Instability in TP53-Deficient Cells. *Cell Reports* **31**, 107465 (2020).
11. Yeh, C.-H., Bellon, M. & Nicot, C. FBXW7: a critical tumor suppressor of human cancers. *Mol Cancer* **17**, 115 (2018).
12. Zehir, A. *et al.* Mutational landscape of metastatic cancer revealed from prospective clinical sequencing of 10,000 patients. *Nat Med* **23**, 703–713 (2017).
13. Hustedt, N. *et al.* A consensus set of genetic vulnerabilities to ATR inhibition. *Open Biology* **9**, (2019).
14. Tycko, J. *et al.* Mitigation of off-target toxicity in CRISPR-Cas9 screens for essential non-coding elements. *Nat Commun* **10**, 4063 (2019).
15. Zhu, L. J., Holmes, B. R., Aronin, N. & Brodsky, M. H. CRISPRseek: A Bioconductor Package to Identify Target-Specific Guide RNAs for CRISPR-Cas9 Genome-Editing Systems. *PLoS ONE* **9**, e108424 (2014).
16. Clouaire, T. *et al.* Comprehensive Mapping of Histone Modifications at DNA Double-Strand Breaks Deciphers Repair Pathway Chromatin Signatures. *Molecular Cell* **72**, 250-262.e6 (2018).
