# Supplementary material for: TP53-dependent toxicity of CRISPR/Cas9 cuts is differential across genomic loci and can confound genetic screening
Source: Nat Commun. 2022 Aug 4;13:4520. doi: 10.1038/s41467-022-32285-1 (PMC9352712; doi:10.1038/s41467-022-32285-1)
Supplement: Supplementary file 2 — Reporting Summary [file 41467_2022_32285_MOESM2_ESM.pdf]

nature portfolio

Corresponding author(s): Fran Supek

Last updated by author(s): Jul 20, 2022

Reporting Summary

Nature Portfolio wishes to improve the reproducibility of the work that we publish. This form provides structure for consistency and transparency in reporting. For further information on Nature Portfolio policies, see our Editorial Policies and the Editorial Policy Checklist.

Statistics

For all statistical analyses, confirm that the following items are present in the figure legend, table legend, main text, or Methods section.

n/a	Confirmed	
☐	☒	The exact sample size (*n*) for each experimental group/condition, given as a discrete number and unit of measurement
☐	☒	A statement on whether measurements were taken from distinct samples or whether the same sample was measured repeatedly
☐	☒	The statistical test(s) used AND whether they are one- or two-sided<br>*Only common tests should be described solely by name; describe more complex techniques in the Methods section.*
☐	☒	A description of all covariates tested
☐	☒	A description of any assumptions or corrections, such as tests of normality and adjustment for multiple comparisons
☐	☒	A full description of the statistical parameters including central tendency (e.g. means) or other basic estimates (e.g. regression coefficient) AND variation (e.g. standard deviation) or associated estimates of uncertainty (e.g. confidence intervals)
☐	☒	For null hypothesis testing, the test statistic (e.g. *F*, *t*, *r*) with confidence intervals, effect sizes, degrees of freedom and *P* value noted<br>*Give P values as exact values whenever suitable.*
☒	☐	For Bayesian analysis, information on the choice of priors and Markov chain Monte Carlo settings
☒	☐	For hierarchical and complex designs, identification of the appropriate level for tests and full reporting of outcomes
☐	☒	Estimates of effect sizes (e.g. Cohen's *d*, Pearson's *r*), indicating how they were calculated

Our web collection on statistics for biologists contains articles on many of the points above.

Software and code

Policy information about availability of computer code

Data collection	No special software was used for collecting the new sequencing data (Illumina HiSeq). We also used previously collected data available from either public databases or other publications as described in the Methods.
Data analysis	MAGeCK-VISPR v0.5.6 was used for alignment of the generated reads to the library, read counting, read count median normalization, quality control, and essentiality analyses.<br>drugZ and BAGEL v2 were employed to validate MAGeCK-VISPR results.<br>HOMER was used to identify DNA motifs enriched in nearby target loci in comparison with background loci.<br>Annovar (v. 2020Jun08) was used to annotate CADD scores for somatic mutations.<br>liftOver was employed for converting genomic coordinates.<br>The remaining analyses were carried out using Python (versions 2.7 and 3.6) and R environments (versions 3.6 and 4.1). Relevant Python packages are mageck-vispr, gdsctools, and random. Relevant R packages are dplyr, devtools, MAGeCKFlute, pROC, ComplexHeatmap, eulerr, Crisprseek, GenomicRanges, rtracklayer, MASS, seqPattern, EMclust, Biostrings, and regionMut (https://github.com/davidmasp/regionMut).<br>Custom Python code for randomization analysis, and custom R function for odds-ratio calculation, are available at https://github.com/mmaalvarez/code_natcom_2022.

For manuscripts utilizing custom algorithms or software that are central to the research but not yet described in published literature, software must be made available to editors and reviewers. We strongly encourage code deposition in a community repository (e.g. GitHub). See the Nature Portfolio guidelines for submitting code & software for further information.

Data

Policy information about availability of data

All manuscripts must include a data availability statement. This statement should provide the following information, where applicable:

- Accession codes, unique identifiers, or web links for publicly available datasets
- A description of any restrictions on data availability
- For clinical datasets or third party data, please ensure that the statement adheres to our policy

New data generated in this study are available in the Supplementary material. We further used the CRISPR screening data from Biayna et al. 2021. We also employed publicly available data as described in the Methods: In brief, we used pan-cancer essentiality data from Achilles and PScore projects, and gene essentiality information from the DEMETER2 project, available through DepMap repository (https://www.depmap.org); CRISPR screening data of TP53-isogenic cell lines was obtained from Brown et al. 2019, Drainas et al. 2020, and from Taipale lab; A list of top-50 TP53 interactor genes was obtained from STRING (https://string-db.org/); Chromatin mark and DNase ChipSeq data was obtained from RoadMap Epigenomics Consortium (http://www.roadmapepigenomics.org/); Copy number data obtained from Levatic et al. (https://doi.org/10.1101/2021.05.19.444811); Gene expression levels obtained from Salvadores et al. 2020; Replication time, CTCF and Cohesin motifs data obtained from Supek and Lehner 2017; Lamin B1 data obtained from genome.ucsc.edu; allele frequency data obtained from gnomAD (Karczewski et al. 2020); CFE (GDSC1) data obtained from Iorio et al. 2016; Mutation data for MSK cohorts from the project GENIE66 v10.1 was obtained from cBioPortal (https://www.cbioportal.org/); A list of validated ATRi-sensitizers was obtained from Hustedt et al. 2019; a list of core-essential and non-essential genes was obtained from Hart et al. 2014 and Hart et al. 2017.

Field-specific reporting

Please select the one below that is the best fit for your research. If you are not sure, read the appropriate sections before making your selection.

- [x] Life sciences
- [ ] Behavioural & social sciences
- [ ] Ecological, evolutionary & environmental sciences

For a reference copy of the document with all sections, see nature.com/documents/nr-reporting-summary-flat.pdf

Life sciences study design

All studies must disclose on these points even when the disclosure is negative.

Sample size	For the main analyses we used 20 CRISPR screening samples from the A549 TP53-isogenic cell line pair, consisting of 77,441 sgRNAs sequenced at an average depth of 39.23M (SD = 1.45M) reads per sample (~500 reads per sgRNA); 14 samples were from Biayna et al. 2021 and the remaining 6 are new data.<br>The Achilles 21Q2 dataset consists of read count data for 74,362 sgRNAs in 856 cell lines.<br>The combined Achilles and Project Score dataset consists of essentiality data for 17,486 genes in 906 cell lines.<br>Drainas et al. 2020 CRISPR screening data consists of 8 samples in which 112,955 sgRNAs were sequenced at an average depth of 57.05M (SD = 40.99M) reads per sample (~500 reads per sgRNA).<br>Taipale CRISPR screening data consists of 4 samples in which 75,077 sgRNAs were sequenced at an average depth of 32.11M (SD = 3.39M) reads per sample (~400 reads per sgRNA).<br>The sequencing depth of these screenings is in compliance with the recommended ~200 reads per sgRNA (Miles, Garippa, and Poirier, 2016. Design, execution, and analysis of pooled in vitro CRISPR/Cas9 screens).
Data exclusions	A549 samples at 3 and 6 days of cell culture were not employed in the analyses, since there had not been enough time for selection to be apparent.<br>Taipale lab's sample replicate R2 was removed due to concerns about its quality.<br>Drainas et al. 2020 samples with 3D condition were not used because of their special growing conditions.
Replication	Untreated and doxycycline-treated A549 cells were always considered independent biological pseudo-replicates of a control sample (i.e. non ATRi-treated), while doxycycline+ATRi treated cells were also considered as another independent pseudo-replicate in some analyses (please see Methods for details). All attempts at replication were successful.
Randomization	Wells with seeded cells were randomly chosen for the corresponding treatments. There were no known covariates during experimental work.<br>Read count normalization was based on either the counts of non-targeting sgRNAs or sgRNAs targeting known non-essential genes, or on the total sequencing depth (please see Methods for details).<br>Regarding the data analysis, we controlled for covariates via negative binomial regression (see Methods).
Blinding	Investigators were not blinded to group allocation during experiments nor during data analyses. All A549 screens were performed and experimentally designed by the same person. The experiments in any comparisons used cells growing in the same conditions.

Reporting for specific materials, systems and methods

We require information from authors about some types of materials, experimental systems and methods used in many studies. Here, indicate whether each material, system or method listed is relevant to your study. If you are not sure if a list item applies to your research, read the appropriate section before selecting a response.

Materials & experimental systems

n/a	Involved in the study
☒	☐ Antibodies
☐	☒ Eukaryotic cell lines
☒	☐ Palaeontology and archaeology
☒	☐ Animals and other organisms
☒	☐ Human research participants
☒	☐ Clinical data
☒	☐ Dual use research of concern

Methods

n/a	Involved in the study
☒	☐ ChIP-seq
☒	☐ Flow cytometry
☒	☐ MRI-based neuroimaging

Eukaryotic cell lines

Policy information about cell lines

Cell line source(s)	A549 cell line was generously provided by the Nebreda laboratory (IRB Barcelona)
Authentication	A549 cell line was authenticated by using an STR profile analysis
Mycoplasma contamination	Cell line tested negative for mycoplasma
Commonly misidentified lines (See ICLAC register)	No commonly misidentified cell lines were used in this study
