# Supplementary material for: TP53-dependent toxicity of CRISPR/Cas9 cuts is differential across genomic loci and can confound genetic screening
Source: Nat Commun. 2022 Aug 4;13:4520. doi: 10.1038/s41467-022-32285-1 (PMC9352712; doi:10.1038/s41467-022-32285-1)
Supplement: Supplementary file 3 — Description of Additional Supplementary Files [file 41467_2022_32285_MOESM3_ESM.pdf]

Title: Supplementary Dataset 1.
Description: Classification of cell lines from the Achilles project based on mutation status. 0 indicates wild-type, 1 indicates mutated phenotype. Source data are provided as a Source Data file.

Title: Supplementary Dataset 2.
Description: p53-related DSB toxicity score for several widely-used CRISPR screening libraries.
